# Associations of cigarette smoking with gray and white matter in the UK Biobank

**DOI:** 10.1038/s41386-020-0630-2

**Published:** 2020-02-07

**Authors:** Joshua C. Gray, Matthew Thompson, Chelsie Bachman, Max M. Owens, Mikela Murphy, Rohan Palmer

**Affiliations:** 1 Department of Medical and Clinical Psychology, Uniformed Services University, 4301 Jones Bridge Rd, Bethesda, MD 20814 USA; 2 Behavioral Genetics of Addiction Laboratory, Department of Psychology at Emory University, 36 Eagle Row, Atlanta, GA 30322 USA; 3 Department of Psychiatry, University of Vermont, 1 South Prospect St., Burlington, VT 05401 USA

**Keywords:** Neuroscience, Human behaviour, Brain, Addiction

## Abstract

Cigarette smoking is associated with increased risk for myriad health consequences including cognitive decline and dementia, but research on the link between smoking and brain structure is nascent. In the current study, we assessed the relationship of cigarette smoking with gray matter (GM) and white matter (WM) in the UK Biobank, controlling for numerous confounding demographic and health variables. We used negative-binomial regression to model the association of cigarette smoking (having ever smoked regularly, cigarettes per day, and duration smoked) with GM and WM (GM *N* = 19,615; WM *N* = 17,760), adjusting for confounders. Ever smoked and duration were associated with smaller total GM volume. Ever smoked was associated with reduced volume of the right VIIIa cerebellum and elevated WM hyperintensity volume. Smoking duration was associated with reduced total WM volume. Regarding specific tracts, ever smoked was associated with reduced fractional anisotropy in the left cingulate gyrus part of the cingulum, left posterior thalamic radiation, and bilateral superior thalamic radiation, and increased mean diffusivity in the middle cerebellar peduncle, right medial lemniscus, bilateral posterior thalamic radiation, and bilateral superior thalamic radiation. This study identified significant associations of cigarette exposure with global measures of GM and WM, and select associations of ever smoked, but not cigarettes per day or duration, with specific GM and WM regions. By controlling for important sociodemographic and health confounders, such as alcohol use, this study identifies distinct associations between smoking and brain structure, highlighting potential mechanisms of risk for common neurological sequelae (e.g., dementia).

## Introduction

Cigarette smoking is the leading cause of preventable death around the world [1]. It is associated with increased risk for psychiatric conditions such as major depressive disorder [2] and alcohol use disorder [3]. Cigarette smoking is also associated with lower processing speed, poorer general cognitive ability, poorer decision-making, and increased impulsivity [4–6]. Furthermore, it is associated with increased risk for cognitive decline and dementia, particularly in older individuals [7, 8]. Globally, it is estimated that 14% of Alzheimer’s disease cases could be attributable to smoking, which represents a significant modifiable risk factor [9]. The link between smoking and Alzheimer’s may occur through the effects of smoking on brain morphometry given that deterioration in gray and white matter is a signature feature of Alzheimer’s and other forms of dementia [10]. Smoking exerts numerous deleterious effects via oxidative stress, inflammation, and atherosclerotic processes that can translate to brain atrophy [11], leading studies to suggest cigarette smoking can accelerate brain aging [12]. Despite this, research on the link between smoking and gray and white matter is nascent.

With regard to smoking and gray matter research, a meta-analysis of 761 individuals who smoke found that smoking was associated with reductions in the volume of the left insula, right cerebellum, left parahippocampus, mediodorsal thalamus, and multiple prefrontal cortical regions [13]. A recent study using a partial sample (total *N* *=* 9631, consisting of individuals who currently smoke *n* = 399, no longer smoke *n* = 3322, and who have never smoked *n* = 5910) from the UK Biobank looking at several cardiovascular risk factors found that a greater number of cigarette pack years (i.e., cigarettes per day divided by 20 and then times the number of years smoked) was associated with reduced total gray matter volume and reduced volume of the thalamus, basal ganglia, hippocampus, and several cortical regions [14]. This body of research offers important preliminary findings on the link between smoking and gray matter. However, most prior studies did not consider multiple indicators of cigarette smoking exposure or account for potential confounding variables such as alcohol use and cardiovascular disease. Including covariates, such as alcohol use, may be particularly important as a recent “mega-analysis” of 2140 individuals with substance use disorders found reduced volume in several regions, but follow-up analyses clarified these effects were primarily related to alcohol dependence with no effects when the analyses were restricted to individuals who currently smoke cigarettes (*n* = 602) [15]. These findings are consistent with a recent study that found gray matter differences between individuals who do and do not smoke were no longer present after adjusting for alcohol use [16].

There have also been several studies linking cigarette smoking to white matter microstructure. The most common measure of white matter microstructure studied to date is fractional anisotropy (FA), a measure of the overall directionality of water diffusion which reflects fiber density, axonal diameter, and myelination. These studies have generally found decreased FA in individuals who smoke [17–20], though there have been some exceptions [21, 22]. Additionally, individuals who smoke have been found to have more white matter lesions (also referred to as hyperintensities) [23, 24]. The study by Cox et al. [19] described above also explored associations of cigarette pack years and white matter, finding associations with less global FA, more global mean diffusivity (MD; another primary measure of white matter microstructure—elevated levels typically indicate reduced structural integrity), and small associations with FA and MD in several individual tracts [14]. Similar to the gray matter research studies, the majority of these studies did not account for potential confounding factors such as alcohol and cardiovascular risk factors.

The association between smoking and brain structure is particularly difficult to establish, as smoking has several common psychiatric, cardiovascular, and demographic risk factors that are also linked to brain morphometry. With regard to psychiatric risk factors, smoking and brain structure are associated with other substance abuse, particularly heavy alcohol use [25, 26]. Indeed, the studies by Mackey et al. [15] and Elbejjani et al. [16] described above both found that the relationship of smoking with gray matter volume was contingent on alcohol use. Furthermore, smoking and brain structure are associated with numerous cardiovascular variables such as hypertension and body mass index (BMI) [14, 27]. Finally, demographic factors such as socioeconomic status, age, and gender are all associated with cigarette smoking and variation in brain morphometry [28–32]. Indeed, a recent methodological study found that including these demographic variables as covariates can have a major impact on the results of brain morphometry studies [33]. Despite these issues of multicollinearity, few studies have examined the association of smoking and brain structure controlling for important confounds such as alcohol use, health, and demographic variables.

The current study aimed to build upon prior research by examining the relationship between gray and white matter reductions and cigarette exposure in the largest sample to date, controlling for numerous confounders. The UK Biobank is one of the largest neuroimaging databases in the world and its database of subjects with MRI data continues to grow [34]; this study analyzed the most comprehensive release of this data to date. Specifically, the present investigation assessed the relationship of cigarette smoking (*ever smoked* [on most or all days for at least 1 year], *cigarettes per day*, and *duration*) with gray and white matter using the UK Biobank cohort (gray matter *N* = 19,615; white matter *N* = 17,760), adjusting for confounders (age, sex, ethnicity, income, education, BMI, alcohol use, cardiovascular risk factors, years since quitting smoking, and global gray and white matter). This study grouped individuals who are currently smoking or formerly smoked into a single group (*ever smoked*) to be consistent with recent studies with the UK Biobank sample including the prior study morphometric by Cox et al. [19] and another that found a stronger casual effect of ever having smoked (current and former smoking) versus smoking intensity on long-term health [35]. Additionally, this study utilized duration and cigarettes per day because duration has been found to be more impactful than a volumetric composite (i.e., pack years) on health outcomes such as lung disease [36, 37]. Prior studies have concluded that incorporating cigarettes per day into a composite with duration may reduce the association between cigarette smoking and health outcomes. We hypothesized that multiple indicators of cigarette exposure would be associated with global brain measures (i.e., reduced total gray and white matter volume and increased white matter hyperintensity volume). We did not hypothesize specific regional associations given our expectation that accounting for confounding variables such as alcohol use and cardiovascular disease would modify and potentially attenuate many prior reported associations.

## Methods and materials

### Materials and procedure

Ethics approval for the UK Biobank study was obtained from the North West Centre for Research Ethics Committee (11/NW/0382) and all participants provided informed consent. At the MRI session, participants provided demographic and health information in response to a series of touchscreen questions. Additionally, a nurse conducted a medical history interview which included self-reported medical diagnoses including neurological conditions/incidents that were used for exclusion (touchscreen questions, consent forms, ethical approval, and other study details are available at: http://www.ukbiobank.ac.uk/key-documents/). Blood pressure was collected twice, moments apart, using an Omron 705IT monitor or a manual sphygmometer if this was unavailable. An average of the two readings was used or if only one reading was present, this single reading was used. Waist and hip measurements were conducted to provide waist to hip ratio and body mass index was calculated as weight (kg)/height^2^ (m).

As per previous UKB studies [14], participants were excluded if they reported any of the following neurological conditions/incidents: dementia, Parkinson’s disease, brain cancer, brain hemorrhage, brain abscess, aneurysm, cerebral palsy, encephalitis, head injury, nervous system infection, head or neurological injury, trauma, stroke, or other chronic degenerative neurological problem (including demyelinating diseases). Following these exclusions, there were 20,760 and 18,789 participants with complete MRI and DTI data, respectively. Additional participants were excluded for missing ethnicity data (MRI *N* = 62, DTI *N* = 58). Finally, participants were excluded for reporting missing or unclear smoking data (i.e., smoking cigarettes on most or all days but then indicating less than 1 cigarette per day, reporting they started and stopped smoking in the same year leading to a duration estimate of 0 years, or endorsing current cigar or pipe smoking but never cigarette smoking; MRI *N* = 1083, DTI *N* = 971). Following exclusions, there were 19,615 subjects with MRI data and 17,760 with DTI data that passed automated and visual quality control (QC) by UK Biobank Imaging group. Missing covariate data were imputed (see below).

### MRI acquisition and processing

MRI data were acquired in a Siemens Skyra 3T scanner using a standard Siemens 32-channel head coil [32]. Briefly, T1-weighted MPRAGE, T2-weighted FLAIR volumes, and diffusion tensor volumes were acquired at 1 × 1 × 1 mm (208 × 256 × 256 field of view [FOV] matrix), 1.05 × 1 × 1 mm (192 × 256 × 256 FOV matrix), and 2 × 2 × 2 mm (104 × 104 × 72 FOV matrix), respectively. The gray and white matter variables utilized in this study were derived from the image-derived phenotypes (IDPs) released by the UK Biobank team (for more details on the processing pipeline and IDP generation for the gray and white matter measures see [34]).

Structural MRI data were processed applying a pipeline to the T1 images that used gradient distortion correction, field of view reduction, registration to the standard atlas, brain extraction, defacing, and finally segmentation. This study utilized the global brain measures and regional gray matter volumes for 139 cortical and subcortical structures. The T2 images were transformed to T1 space and processed through a similar pipeline to the T1 images. Total volume of white matter hyperintensities was calculated from both T1 and T2 image data. Of note, we included white matter hyperintensity volume in the DTI subset of analyses because there were 1201 fewer subjects with valid T1 and T2 data than with T1 data (used in the MRI analyses), it was included as a covariate in the DTI analyses, and grouping it with the DTI data only resulted in 43 additional subjects DTI data being excluded. Diffusion MRI (dMRI) data were corrected for eddy currents and head motion, had outlier-slices corrected, and then were corrected for gradient distortion. The output from this process was run through two complementary analysis pipelines, one using probabilistic tractography and one using tract-skeleton processing. This study utilized the 27 FA and MD tracts generated from the probabilistic tractography analysis.

### Analyses

Imputation of missing values was conducted utilizing multivariate imputation by chained equations implemented in the R package “mice”. Multivariate imputation by chained equations is a robust method for imputation and recommended above other methods such as mean imputation or complete case analysis which can bias results [38]. For example, there are some data which are likely to not be missing at random, such as unwillingness to report household income and thus excluding these individuals could bias the sample. Given the possibility that some variables, particularly household income, are not missing completely at random, the “MNAR” (i.e. missing not at random) mechanism was utilized. We imputed missing data for household income (MRI: 9.2%, DTI: 9.3%), systolic blood pressure (MRI: 4.1%, DTI: 4.3%,) diastolic blood pressure (MRI: 4.1%, DTI: 4.3%), body mass index (MRI: 2.2%, DTI: 2.3%), waist-hip ratio (MRI: 1.9%, DTI: 2.0%), alcohol consumption (MRI: 0.8%, DTI: 0.8%), and college degree (MRI: 0.3%, DTI: 0.3%). Variables exhibiting a skew of more than ±2 were log or square root transformed to improve their distribution (whichever transformation most improved the distribution was utilized). White matter hyperintensity volume was log-transformed and alcohol use was square root transformed to correct for a positive-skew distribution.

We assessed for associations of *ever smoked* (1 = current or former smoking on most or all days, 0 = never smoking on most or all days), *cigarettes per day* (present if they are currently smoking or past if they are no longer smoking), and smoking *duration* (age at the time of data collection minus the year they started smoking if they are currently smoking, and the year they stopped smoking minus the year they started smoking if they are no longer smoking) with gray matter and white matter controlling for age, sex, ethnicity, income, college degree, alcoholic drinks per month, body mass index, waist-hip ratio, diastolic and systolic blood pressure, and *x*, *y*, *z* position in the scanner. *Cigarettes per day* and *duration* also controlled for years since quitting smoking (i.e., age when stopped smoking on most days minus age of initiation). Associations with individual gray matter regions additionally adjusted for total gray matter volume and individual white matter tract analyses adjusted for total white matter volume and white matter hyperintensity volume.

The smoking count data (*cigarettes per day*, *duration*) exhibited zero-inflation and data overdispersion (~78% of the sample never regularly smoked cigarettes and therefore were considered never smokers, and the variance of *cigarettes per day* and *duration* were multiple times greater than the mean) as is typical [39]. Thus a hurdle negative-binomial regression was utilized to account for these properties in our sample. These analyses included a prediction of *ever smoked* and estimation of *cigarettes per day* among those with >0 cigarettes per day, and estimation of *duration* (rounded to the nearest whole number) among those with > 0 years of smoking. Statistical analyses utilized the R package ‘pscl’.

We tested the associations of *ever smoked*, *cigarettes per day*, and *duration* with total gray matter, white matter, and white matter hyperintensity volume; with the volume of 139 cortical and subcortical regions; and with 54 white matter tracts (27 FA and 27 MD). Separate models were run for each region. We conducted separate false-discovery rate (FDR) corrections for the 3 global brain measures, 139 gray matter regions, and 54 white matter regions [40]. For descriptive purposes, we also conducted bivariate correlations among the smoking exposure variables, covariates, and global brain measures. We have provided our scripts for conducting our analyses online (https://github.com/CNPsyLab/UKB-Smoking) to facilitate replication and extension of these findings with additional participants (https://imaging.ukbiobank.ac.uk/) and novel analyses.

## Results

### Gray matter analyses

The final sample for gray matter analyses after exclusions was 19,615 (Table 1). Participants were 62.9 years old (range 44.6–80.1), 53.5% female, and comprised of 369 (1.9%) current, 3873 (19.7%) former, and 15,373 (78.4%) never smoking individuals. Smoking exposure variables were significantly associated with the majority of the covariates in bivariate correlations; however, among individuals who *ever smoked*, *cigarettes per day* and *duration* were non-significantly associated (Table S1).

**Table 1:** Participant characteristics.

	Gray matter analyses		White matter analyses	
Characteristic	N / M	%	N / M	%
**Total** ***N***	19615		17760	
*Smoking variables*				
Smoking status (current; former; never)^a^	369; 3873; 15373	1.9%; 19.7%; 78.4%	312; 3460; 13988	1.8%; 19.5%; 78.6%
Cigarettes per day^b^	17.1 (SD = 8.9)		17.0 (SD = 8.9)	
Duration^b^	22.9 (SD = 12.9)		22.7 (SD = 12.8)	
*Covariates*				
Age	62.9 (SD = 7.5)		62.9 (SD = 7.4)	
Female	10503	53.5%	9565	53.9%
Ethnicity (non-White)	543	2.8%	473	2.7%
Income	£31000-£51,999^c^ (IQR: £18,000-£100,000)		£31,000-£51,999^c^ (IQR: £18,000-£100,000)	
College degree	9287	47.3%	8403	47.3%
Body mass index	26.6 (SD = 4.4)		26.5 (SD = 4.4)	
Waist-hip ratio	0.87 (SD = 0.08)		0.86 (SD = 0.08)	
Systolic blood pressure	136.9 (SD = 17.9)		136.9 (SD = 17.9)	
Diastolic blood pressure	78.7 (SD = 10.0)		78.6 (SD = 10.0)	
Alcohol drinks per month^d^	29.9 (SD = 32.9)		29.8 (SD = 32.8)	
Years since quitting smoking^e^	23.7 (SD = 14.4)		23.9 (SD = 14.3)	
*MRI covariates*^f^				
Total gray matter volume (mm^3^)	617000 (SD = 55621.9)			
Total white matter volume (mm^3^)			548900 (SD = 61857.2)	
White matter hyperintensity volume^g^ (mm^3^)			4357 (SD = 5573.8)	

*N* number, *M* mean, *IQR* interquartile range, *SD* standard deviation

^a^Smoking on most or all days for at least 1 year was used to define current and former smoking

^b^Only includes individuals who reported current or former smoking

^c^Median

^d^Refers to current drinks per month

^e^ =0 for individuals currently smoking and does not include individuals who have never smoked

^f^Additional covariates included X, Y, Z position in the scanner

^g^Values reported are prior to log-transformation

**Fig. 1:**
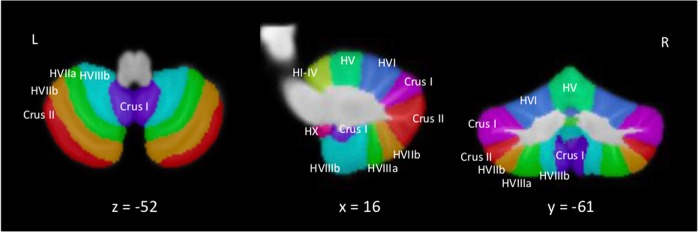
Cerebellar atlas overlaid on a group average of 20 healthy subjects. These images are derived from the publically available viewer (http://www.diedrichsenlab.org/imaging/AtlasViewer/viewer.html; [50]) and thus are not from the UK Biobank sample.

In the primary analyses, *ever smoked* and *duration* were significantly associated and *cigarettes per day* was marginally associated (i.e., *p* *<* 0.05, but not exceeding the threshold of multiple comparison correction) with smaller total gray matter volume. *Ever smoked* was associated with reduced volume of the right VIIIa cerebellum (the cerebellar atlas is depicted in Fig. 1), whereas *cigarettes per day* and *duration* were not associated with any individual regions after FDR correction. Only regions for which there is a significant association with at least 1 of the smoking variables are depicted in Table 2. All tested regions are depicted in Table S2.

**Table 2:** Significant associations of smoking variables with gray matter volume.

	Cigarettes per day			Duration			Ever smoked		
Region	β	*p*	*q*	β	*p*	*q*	β	*p*	*q*
Total gray matter volume	−0.021	0.023	0.068	−**0.012**	**0.004**	**0.012**	−**0.094**	**0.00003**	**4.50E−05**
VIIIa cerebellum (R)	0.009	0.320	0.772	0.002	0.631	0.911	−**0.089**	**0.0001**	**0.010**

Standardized beta coefficients (β) and *p* values are reported for hurdle negative-binomial regression models of smoking variables predicting gray matter volume. Bold indicates FDR significance (*q* < 0.05). Full results are reported in Table S2.

### White matter analyses

The final sample for white matter analyses after exclusions was 17,760 (Table 1). Participants were 62.9 age (range 45.2–80.7), 53.9% female, and comprised of 312 (1.8%) current, 3460 (19.5%) former, and 13,988 (78.6%) never smoking. Smoking exposure variables were significantly associated with the majority of the covariates in bivariate correlations; however, among individuals who *ever smoked*, *cigarettes per day* and *duration* were non-significantly associated (Table S1).

**Fig. 2:**
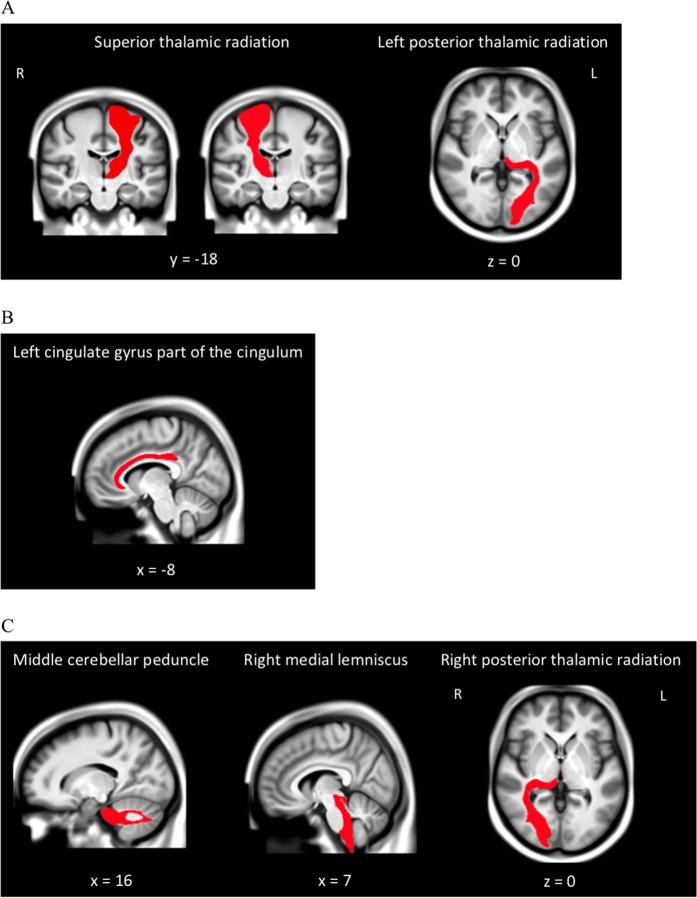
Group-average structural MRI with significant white matter tracts overlaid, estimated from a subset of UK Biobank study participants (*N* = 4500). Images derived from the publically available viewer (https://www.fmrib.ox.ac.uk/ukbiobank/group_means/index.html). Panel A depicts the tracts that were significant in both the fractional anisotropy (FA) and mean diffusivity (MD) analyses. Panel B depicts the tract that was significant in the FA analyses. Panel C depicts the tracts that were significant in the MD analyses.

In the primary analyses, e*ver smoked* was significantly associated with elevated white matter hyperintensity volume and *duration* was significantly associated with reduced total white matter volume. With regard to specific tracts, *ever smoked* was associated with reduced FA in the left cingulate gyrus part of the cingulum, left posterior thalamic radiation, and bilateral superior thalamic radiation and increased MD in the middle cerebellar peduncle, right medial lemniscus, bilateral posterior thalamic radiation, and bilateral superior thalamic radiation. Associations of *cigarettes per day* and *duration* with MD and FA of specific tracts did not survive FDR correction. Only regions for which there is a significant association with at least one of the smoking variables are depicted in Table 3 and Fig. 2. All tested regions are depicted in Table S3.

**Table 3:** Significant associations of smoking variables with white matter metrics.

	Cigarettes per day			Duration			Ever smoked		
Region	β	*p*	*q*	β	*p*	*q*	β	*p*	*q*
**Total white matter volume**	−0.019	0.051	0.076	−**0.009**	**0.028**	**0.042**	0.009	0.713	0.713
**White matter hyperintensity**	0.011	0.235	0.235	0.001	0.727	0.727	**0.124**	**5.89E−08**	**1.77E−07**
*Fractional anisotropy*									
Cingulate gyrus part of cingulum (L)	−0.001	0.916	0.985	0.002	0.570	0.905	−**0.055**	**0.004**	**0.025**
Posterior thalamic radiation (L)	0.003	0.722	0.985	0.006	0.148	0.443	−**0.059**	**0.004**	**0.025**
Superior thalamic radiation (L)	−0.006	0.450	0.985	0.002	0.508	0.832	−**0.052**	**0.006**	**0.033**
Superior thalamic radiation (R)	0.000	0.985	0.985	−0.001	0.823	0.963	−**0.055**	**0.004**	**0.025**
*Mean diffusivity*									
Middle cerebellar peduncle	0.006	0.464	0.985	−0.004	0.270	0.539	**0.070**	**0.0004**	**0.007**
Medial lemniscus (R)	−0.004	0.641	0.985	0.001	0.685	0.963	**0.060**	**0.002**	**0.022**
Posterior thalamic radiation (L)	−0.007	0.405	0.985	−0.007	0.074	0.413	**0.072**	**0.001**	**0.010**
Posterior thalamic radiation (R)	−0.015	0.080	0.985	−0.001	0.769	0.963	**0.091**	**0.00002**	**0.001**
Superior thalamic radiation (L)	0.006	0.473	0.985	0.001	0.785	0.963	**0.071**	**0.002**	**0.018**
Superior thalamic radiation (R)	−0.001	0.931	0.985	0.003	0.502	0.832	**0.094**	**0.0001**	**0.002**

Standardized beta coefficients (β) and *p* values are reported for hurdle negative-binomial regression models of smoking variables predicting white matter metrics. Bold indicates FDR significance (*q* < 0.05). Full results are reported in Table S3.

## Discussion

In the largest sample to date, controlling for numerous potential confounders, we found significant associations of cigarette exposure with global measures of gray and white matter. This underscores the widespread effects of smoking on brain structure after accounting for alcohol use, cardiovascular disease, and other health risk factors. Furthermore, we found select associations of *ever smoked*, but not *cigarettes per day* or *duration*, with specific gray and white matter regions.

The significant associations of *ever smoked* and *duration* with smaller total gray matter volume is consistent with prior studies [13, 14]. Whole brain atrophy is a signature feature of neurodegeneration [10], and thus may be one way in which smoking increases risk for neurocognitive disorder. The marginally significant association between smoking *duration* and total white matter volume, suggests only a weak relationship after accounting for covariates. The largest association identified in this study was of *ever smoked* with increased white matter hyperintensity volume. White matter hyperintensities are thought to serve as an indicator of microvascular damage to the brain and have been described as a general marker of brain frailty (for a review see [41]). White matter hyperintensities are associated with cognitive impairment, doubled risk for dementia, and tripled risk for stroke [41]. Indeed, a recent study using a subset of the UK Biobank sample found that the *APOE* e4 genotype, a well-established risk factor for Alzheimer’s disease, is linked to increased white matter hyperintensity volume [42]. Thus increased white matter hyperintensity volume may be a contributing factor to the increased risk for dementia and Alzheimer’s disease among people who smoke, particularly given there is a thought to be dose-dependent association between white matter hyperintensity volume and clinical outcomes [43, 44]. These associations between cigarette exposure and global brain measures are consistent with prior studies of the effects of smoking on brain morphometry that did not control for alcohol use and cardiovascular disease [14, 23, 24], further establishing a robust association between cigarette exposure and widespread gray matter structural integrity and white matter hyperintensity volume.

With regard to the association between *ever smoked* and reduced volume of the right VIIIa cerebellum, the right cerebellum has been previously linked to smoking in a meta-analysis [13]. Recent research indicates that the cerebellar lobule VIIb/VIIIa is implicated in a variety of cognitive tasks including visual attention and working memory [45, 46]. Furthermore, the cerebellum has recently become recognized as relevant to addictive processes, suggesting it serves as an intermediary between motor and reward, motivation, and cognitive control systems [47, 48]. Although we identified fewer specific regional associations than many prior studies found [13, 14], this was expected, as our investigation included numerous demographic and health factors that co-occur with smoking and are linked to brain morphometry. Additionally, to account for the large number of comparisons in this study we used a stringent multiple comparison correction, which may have obscured some very small effects that contribute to the global differences in gray matter volume. However, given that no individual regional gray matter association had an effect size as large as the associations of total gray matter volume with *ever smoked* and *duration*, it suggests that smoking may exert primarily a global effect on gray matter.

*Ever smoked* was also associated with reduced FA in the left cingulate gyrus part of the cingulum, left posterior thalamic radiation, and bilateral superior thalamic radiation as well as increased MD in the middle cerebellar peduncle, right medial lemniscus, superior thalamic radiation, and bilateral posterior thalamic radiation. Higher levels of FA are an indicator of axonal density and integrity while higher levels of MD are broadly indicative of reduced structural integrity [49], suggesting smoking is linked to poorer overall white matter health on multiple measures. The association of cerebellar gray and white matter with smoking merits further inquiry. The posterior and superior thalamic radiation associations are broadly consistent with a previous finding that the atrophy of these pathways may potentially be due to the disproportionate negative effects of environmental factors on these tracts [50]. The cingulum and medial lemniscus have been linked to other modifiable risk factors such as BMI and physical activity [51] suggesting they are tracts whose integrity is potentially malleable in response to a variety of behavioral factors. Although we focused on FA and MD in this study due to their broad significance in overall white matter integrity, their use in most prior research (e.g., [14]), and to maintain a manageable FDR correction, it will be important for future research studies to explore the links of smoking with other white matter metrics (e.g., axial and radial diffusivity).

The majority of significant associations found in this study, including the robust association with white matter hyperintensity volume, were with *ever smoked*, rather than *cigarettes per day* or *duration*. This is consistent with a recent finding that ever smoking is more strongly associated with death due to cardiovascular reasons than smoking intensity [35]. There are numerous potential reasons for the stronger link with *ever smoked*. First, this study utilized a retrospective self-report, thus there is potential for recall bias in the *cigarettes per day* and *duration* of smoking variables. Additionally, average cigarettes per day does not capture fluctuations in use, and self-reported cigarettes per day has been shown to correlate poorly with biochemical assessments of smoking exposure [52]. Similarly, duration of smoking does not take into account the level of actual smoke exposure and fails to account for gaps in time where they temporarily stopped or significantly reduced smoking. Finally, the *ever smoked* analyses utilized the whole sample, whereas the continuous *cigarettes per day* and *duration* analyses only included individuals reporting current or former smoking—about one fifth of the total sample. Thus our findings highlight the limitations of various smoking exposure indices and caution against drawing conclusions from a single indicator of smoking exposure.

There are some limitations to the present investigation. First, the data are cross-sectional and thus we are unable to disentangle the causality and timing of these links between smoking and brain structure identified here. It is possible that the variation in brain morphometry may in part be a precipitating factor for initiating smoking. However, it is unlikely that white matter hyperintensity volume precipitate smoking given they typically show up in older age [53] after most have already begun smoking. Additionally, while we excluded participants with neurological conditions and incidents to eliminate potential confounding from these neurodegenerative processes, future research will need to test for a direct link between smoking, brain morphometry, and neurological sequelae. Second, the UK Biobank imaging sample tends to consist of individuals from a higher socioeconomic status background than the general population, potentially limiting generalizability [54]. Furthermore, the age range studied here is in the early-to-mid 60s on average and thus the findings may not generalize to significantly older or younger populations.

Despite these limitations, this study assessed the link between multiple cigarette exposure variables and brain structure in the largest sample to date, accounting for numerous confounds typically not accounted for in prior research. We found significant associations between smoking exposure, namely *ever smoked*, and global brain measures and individual regional measures. These findings inform our understanding of the connections between smoking and variation in brain structure and clarify potential mechanisms of risk for common neurological sequelae (e.g., cognitive decline, dementia).

## Funding and disclosure

This project was supported by a grant from the National Institute on Drug Abuse awarded to Dr. Palmer (R01DA04742). The authors have no conflicts to declare. This research has been conducted using the UK Biobank Resource under Application Number 31187.

## Disclaimer

The opinions and assertions expressed herein are those of the authors and do not necessarily reflect the official policy or position of the Uniformed Services University or the Department of Defense.

## Supplementary information

Supplementary Materials
